# Brain Health Research in PubMed/MEDLINE: A Bibliometric Analysis of Thematic Structure and Health-Economic Terminology with a Conceptual Performance-Management Framework

**DOI:** 10.3390/brainsci16090967

**Published:** 2026-09-13

**Authors:** Ioannis Ch. Lampropoulos, Georgia Xiromerisiou

**Affiliations:** 1 Department of Business Administration, University of Patras, 26504 Patras, Greece; 2 Mindful Mind Institute of Preventive Neurology and Brain Health, 55133 Thessaloniki, Greece; georgiaxiromerisiou@gmail.com; 3 English Medical Program, School of Medicine, Faculty of Health Sciences, Aristotle University of Thessaloniki, 54124 Thessaloniki, Greece

**Keywords:** brain health, bibliometric analysis, VOSviewer, performance management, neurodegeneration, cost-effectiveness, neuroinflammation, biomarkers, machine learning, keyword co-occurrence

## Abstract

**Highlights:**

**What are the main findings?**

- Brain health is an interdisciplinary field with a differentiated thematic structure; the independently retrieved recent-period network prominently represented topics such as neuroinflammation, blood-based biomarkers, and machine-learning applications.
- Within the analyzed PubMed/MEDLINE corpus, health-economic concepts occurred at relatively low frequencies and showed limited prominence in the primary keyword co-occurrence network; targeted analysis of the complete dataset nevertheless identified several such concepts.

**What are the implications of the main findings?**

- The limited prominence of health-economic concepts within the analyzed PubMed/MEDLINE corpus highlights the need for further research examining whether emerging brain health diagnostic, preventive, and monitoring strategies translate into measurable health-system and societal value.
- A conceptual three-axis framework linking disease-cost assessment, the economic evaluation and return on investment of prevention, and population-level brain health performance indicators may inform future research on evidence-based resource allocation and health-system planning.

**Abstract:**

Background/Objectives: Brain health is an interdisciplinary field; however, the prominence of health-economic concepts within the PubMed/MEDLINE literature explicitly retrieved using the term ‘brain health’ has not been systematically characterized. The present study aimed to map the scientific landscape of the retrieved brain health literature and to examine the representation and network prominence of health-economic concepts within this corpus. Methods: Two separate PubMed/MEDLINE searches were conducted using the exact search expression “brain health”. The overall search was performed on 8 April 2026 and yielded an analytical corpus of 18,306 publications (1993–2026), while a separate search on 9 May 2026, restricted by publication year to 2023–2026, yielded the recent-period analytical corpus (*n* = 10,908). Bibliometric analyses were performed using VOSviewer (version 1.6.20), including keyword co-occurrence analysis and thesaurus-based term standardization. Results: The overall keyword co-occurrence analysis identified five principal thematic clusters: neurobiological mechanisms (*n* = 117 items), public health and lifestyle (*n* = 67), neurodegeneration and biomarkers (*n* = 54), neuroimaging and vascular factors (*n* = 37), and neurophysiology and interventions (*n* = 25). The independently retrieved recent-period corpus showed a seven-cluster thematic structure, with prominent topics including neuroinflammation, blood-based biomarkers, and machine-learning applications. The targeted analysis identified health-economic concepts in the complete dataset, including cost–benefit analysis (*n* = 37), cost of illness (*n* = 28), cost-effectiveness (*n* = 24), and healthcare costs (*n* = 21), but these concepts showed relatively low frequency and limited prominence in the principal co-occurrence network. These findings indicate that health-economic concepts are present within the analyzed PubMed/MEDLINE corpus but show relatively low frequency and limited prominence in the principal keyword co-occurrence network. Conclusions: Within the PubMed/MEDLINE corpus analyzed, health-economic concepts showed relatively low frequency and limited prominence in the principal keyword co-occurrence network. Because the analysis was restricted to PubMed/MEDLINE and used the exact search expression ‘brain health’, these findings characterize the retrieved corpus and should not be interpreted as evidence of a general disconnect between brain health and health economics across the broader multidisciplinary literature. Informed by the bibliometric findings and the broader literature discussed in the manuscript, a conceptual three-axis framework is subsequently proposed, comprising: (1) disease-cost assessment, (2) economic evaluation and return-on-investment assessment of preventive interventions, and (3) population-level brain health performance indicators.

## 1. Introduction

The definition of brain health remains heterogeneous across scientific fields [1]. In one of the most comprehensive conceptual analyses of the term, brain health is defined as the ability of the brain to optimally adapt to internal and external conditions through cognitive and emotional responses throughout life, resulting in sustainable positive changes in brain structures and functional properties [1]. In line with this approach, Chen et al. [2] formulated a working definition of brain health as a lifelong, multidimensional and dynamic state consisting of cognitive, emotional and motor dimensions, supported by physiological processes and influenced by eco-biopsychosocial determinants.

From a public health perspective, brain health extends beyond the absence of neurological or psychiatric disease. It includes the cognitive, sensory, socio-emotional, behavioral, and motor functions that enable individuals to realize their potential throughout life [3]. It is therefore closely connected not only to physical and mental health, but also to independence, social participation, productivity, creativity, and overall well-being [4]. This broader understanding places brain health at the intersection of neuroscience, public health, preventive medicine, social policy, and health-system planning.

In particular, the total societal cost of Alzheimer’s disease increases by at least 50% between successive stages of severity, with care by non-professional caregivers accounting for approximately half of the total cost regardless of region [5]. For Parkinson’s disease, annual healthcare resource use increases threefold in advanced stages in the US and Europe, with hospitalizations, medication and indirect costs being the main cost drivers [6]. At the macroeconomic level, the total economic burden of Alzheimer’s disease in the US was $344 billion in 2020 and is projected to exceed $3 trillion by 2060 [7]. Similarly, in China, the cost of dementia management is expected to increase from $22.8 billion in 2010 to over $372 billion by 2050, representing 0.53% of GDP [8]. Evidence from countries of the Organisation for Economic Co-operation and Development (OECD), based on dynamic panel analysis, shows that the rising incidence of neurological disorders is associated with both higher healthcare expenditure and reduced economic output, highlighting their dual economic burden [9].

Physical activity interventions implemented before or during the early stages of cognitive decline have been shown to be cost-effective in reducing the burden of dementia [10], while non-pharmacological interventions—particularly cognitive stimulation, case management and vocational rehabilitation therapy—show strong evidence of cost-effectiveness [11]. At the population level, multisectoral prevention interventions are estimated to be cost-effective and cost-efficient, especially when targeting middle-aged individuals with modifiable risk factors [12], while community interventions that address modifiable risk factors—such as smoking, physical inactivity and low educational level—are shown to be highly cost-effective or even cost-saving [13].

An important example of the translation of this evidence into policy is the Scottish Model of Brain Health Services. This national initiative is based on risk reduction, early detection, public education, and a life-course approach to the prevention of neurodegenerative diseases [14].

Brain health is assessed using heterogeneous approaches. A review of the available literature identified 479 distinct assessment methods, most of which had been used only once, highlighting the absence of a widely accepted measurement framework [4]. Current approaches include neuroimaging techniques, such as structural magnetic resonance imaging, functional magnetic resonance imaging, and diffusion tensor imaging. Other methods include cognitive assessments of memory, attention, and executive function; blood-based and inflammatory biomarkers; and measures of mental health, lifestyle, and social functioning [15].

Systems such as the BABRI Brain Health platform have demonstrated strong potential for community-based screening, with promising sensitivity and specificity for the detection of mild cognitive impairment [16]. Flexible sensors, wearable technologies, and artificial intelligence can integrate multiple physiological and behavioral parameters in real time, creating opportunities for earlier detection, continuous monitoring, and more personalized intervention [17].

Brain–computer interfaces (BCIs) have potential applications in neurological rehabilitation, assistive technologies, and cognitive assessment, training, and monitoring [18,19]. However, broader implementation is constrained by technical and usability limitations and, depending on the BCI approach, by concerns related to invasiveness, safety, privacy, informed consent, and accessibility [18]. Further research is needed to establish validated and cost-effective implementation models while addressing these ethical and practical considerations [19].

Despite the substantial clinical and epidemiological literature on brain health, the representation of health-economic concepts within the PubMed/MEDLINE literature explicitly retrieved using the term “brain health” has not been systematically characterized bibliometrically. The present study addresses this specific question by mapping the thematic structure of the retrieved corpus and examining the representation and network prominence of health-economic concepts within it. For this purpose, 18,306 publications retrieved from PubMed/MEDLINE for 1993–2026 were analyzed using VOSviewer (version 1.6.20). The study addresses the following research questions:

1. What are the principal thematic clusters represented within the PubMed/MEDLINE corpus retrieved using the exact search expression ‘brain health’?
2. What thematic structure characterizes the independently retrieved recent-period (2023–2026) corpus, and which topics show greater recency based on average publication year?
3. To what extent are health-economic concepts represented within the PubMed/MEDLINE corpus retrieved using the exact search expression ‘brain health’?
4. How can the bibliometric findings, considered together with the broader literature discussed in the manuscript, inform the development of a conceptual framework integrating disease-cost assessment, return-on-investment analysis, and population-level brain health indicators?

## 2. Materials and Methods

### 2.1. Search Strategy and Bibliographic Dataset

The overall analytical approach was consistent with previous bibliometric studies using VOSviewer (version 1.6.20) to examine the relationship between medical research and broader thematic or managerial dimensions [20].

**Figure 1:**
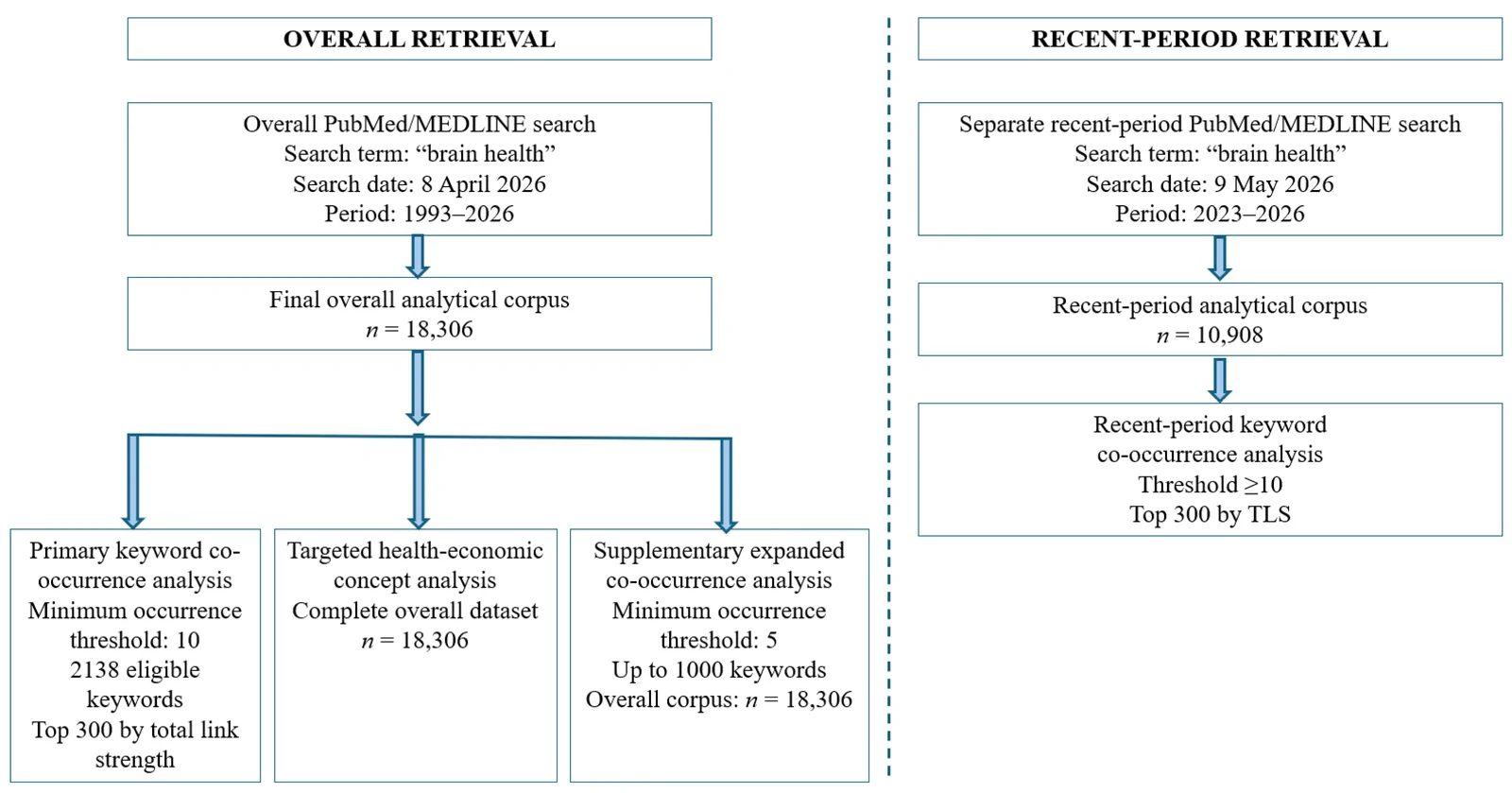
Flow diagram of bibliographic dataset identification and allocation to the four bibliometric analyses. The overall PubMed/MEDLINE search was conducted on 8 April 2026 and yielded the final overall analytical corpus (1993–2026; *n* = 18,306), which was used for the primary keyword co-occurrence analysis, the targeted health-economic concept analysis, and the supplementary expanded co-occurrence analysis. A separate PubMed/MEDLINE search conducted on 9 May 2026 generated the recent-period analytical corpus (2023–2026; *n* = 10,908), which was used for the recent-period keyword co-occurrence analysis. For both analytical periods, 2026 represents a partial publication year because the respective searches were completed before the end of 2026.

A structured bibliographic search was conducted in PubMed/MEDLINE using the exact search expression “brain health”, entered without an explicit PubMed field tag and without additional search filters. The overall search was performed on 8 April 2026 and covered records indexed in PubMed from 1993 to the search date. No publication-type, language, study-design, population, or other search filters were applied to the overall search. The original PubMed bibliographic export files retained from the search are provided as Supplementary Source Files to support transparency and reproducibility. The documented PubMed/MEDLINE search strategy for both the overall and recent-period searches is provided in Supplementary Source File S11. The final analytical corpus used for the overall bibliometric analysis comprised 18,306 publications. The bibliographic dataset was subsequently imported into VOSviewer version 1.6.20 for bibliometric analysis [21]. The identification of the bibliographic datasets and their allocation to the respective analyses are summarized in Figure 1. Annual publication counts for the overall analytical corpus are provided in Supplementary Table S2.

To examine the contemporary structure of the field, a separate PubMed/MEDLINE search was conducted on 9 May 2026 using the exact search expression “brain health”, entered without an explicit PubMed field tag. No additional topical, article-type, language, or other search filters were applied; the retrieval was limited only by publication year to the period 2023–2026. This recent-period search constituted an independently retrieved dataset rather than a publication-year subset of the overall analytical corpus. The final recent-period analytical corpus comprised 10,908 records and was analyzed using the same principal VOSviewer parameters as the overall dataset. Publications from 2026 represent a partial publication year.

### 2.2. Eligibility, Screening, and Duplicate Handling

Records were eligible for inclusion if they were retrieved by the specified PubMed/MEDLINE search strategy and contained bibliographic information sufficient for inclusion in the bibliometric dataset. No manual screening based on study design, population, intervention, language, or article type was performed, because the objective was to characterize the complete bibliographic corpus retrieved by the predefined search strategy rather than to conduct a systematic review of individual study findings. Accordingly, no reviewer-based title/abstract eligibility screening was undertaken. Because the bibliographic corpus originated from a single PubMed/MEDLINE search rather than from the merging of multiple databases, no separate formal database-level de-duplication procedure was documented. The records were exported from the same PubMed search in successive batches and combined during bibliographic data preparation. A retrospective audit of the retained source files identified 18,309 records with unique PMIDs in the original PubMed export batches. Of these, three records had titles duplicated elsewhere in the export and were represented only once in the final bibliographic dataset, yielding a final analytical corpus of 18,306 records entering VOSviewer.

### 2.3. Bibliometric Analysis

Before constructing the bibliometric networks, a customized thesaurus file was developed to improve the consistency and interpretability of the data. The thesaurus was used to combine synonymous terms, spelling variants, abbreviations, and singular or plural forms under a single standardized label. It was also used to exclude duplicate, overly general, or non-informative terms that did not contribute meaningfully to the thematic interpretation of the network, such as broad methodological expressions and selected demographic descriptors. The customized thesaurus used in the analysis is provided as Supplementary Source File S6 to support methodological transparency and reproducibility.

Then, a keyword co-occurrence analysis was performed. This method examines how frequently keywords appear together within the same publications and uses these relationships to identify groups of closely related research topics. In this analysis, the term “keyword” refers to the terms extracted from the bibliographic records and included by VOSviewer in the co-occurrence analysis after application of the customized thesaurus file.

The full counting method was applied, meaning that each occurrence of a keyword and each co-occurrence between two keywords contributed fully to the network. A minimum threshold of 10 occurrences was established for keyword inclusion. Of the 56,210 keywords identified in the dataset, 2138 met this threshold. These terms were ranked according to their total link strength, and the 300 most strongly connected keywords were retained for network visualization and cluster analysis [22,23,24]. The minimum threshold of 10 occurrences was applied to exclude very infrequent terms that would contribute limited information to the principal co-occurrence structure. The subsequent restriction to the 300 terms with the highest total link strength was applied as a network-visualization and interpretability criterion rather than as an eligibility criterion for the underlying bibliographic corpus. Because ranking by total link strength may preferentially retain highly connected terms and exclude less strongly connected concepts, the representation of health-economic terminology was additionally examined in the complete exported dataset independently of this 300-term restriction.

The analysis was conducted in VOSviewer version 1.6.20 using the full counting method. The minimum occurrence threshold and term-selection procedure are reported above. Additional normalization and clustering parameters, including any random seed, are not reported because the retained analytical files do not provide a reliable record of the exact values used, and these settings were not independently documented at the time of the original analysis.

The bibliometric map and its associated data were exported in VOSviewer JSON format [25]. Quantitative information was subsequently extracted for each keyword, including its number of occurrences, total link strength, cluster assignment, and average publication year. The average publication year was used as an indicator of the relative recency of each research topic. These data were used to support the interpretation of the thematic clusters and are presented in Tables 2 and 3.

To assess the representation of health-economic concepts independently of the network-selection procedure, a targeted supplementary analysis was conducted on the complete exported dataset before restriction to the 300 keywords with the highest total link strength. A predefined set of health-economic concepts was selected a priori by the authors on the basis of their relevance to the study objective and their established use in health-economic evaluation terminology. The concepts examined included cost-effectiveness, cost–benefit analysis, cost of illness, economic evaluation, economic burden, healthcare costs and expenditures, quality-adjusted life years (QALYs), cost–utility analysis, health economics, and return on investment. Lexical variants differing in capitalization, hyphenation, pluralization, and indexing format were consolidated under the corresponding concept. The number of unique publications containing each concept was then determined. This analysis was used to distinguish true absence from limited prominence resulting from the keyword-selection procedure used for network visualization.

## 3. Results

### 3.1. Keyword Co-Occurrence Network Analysis

**Figure 2:**
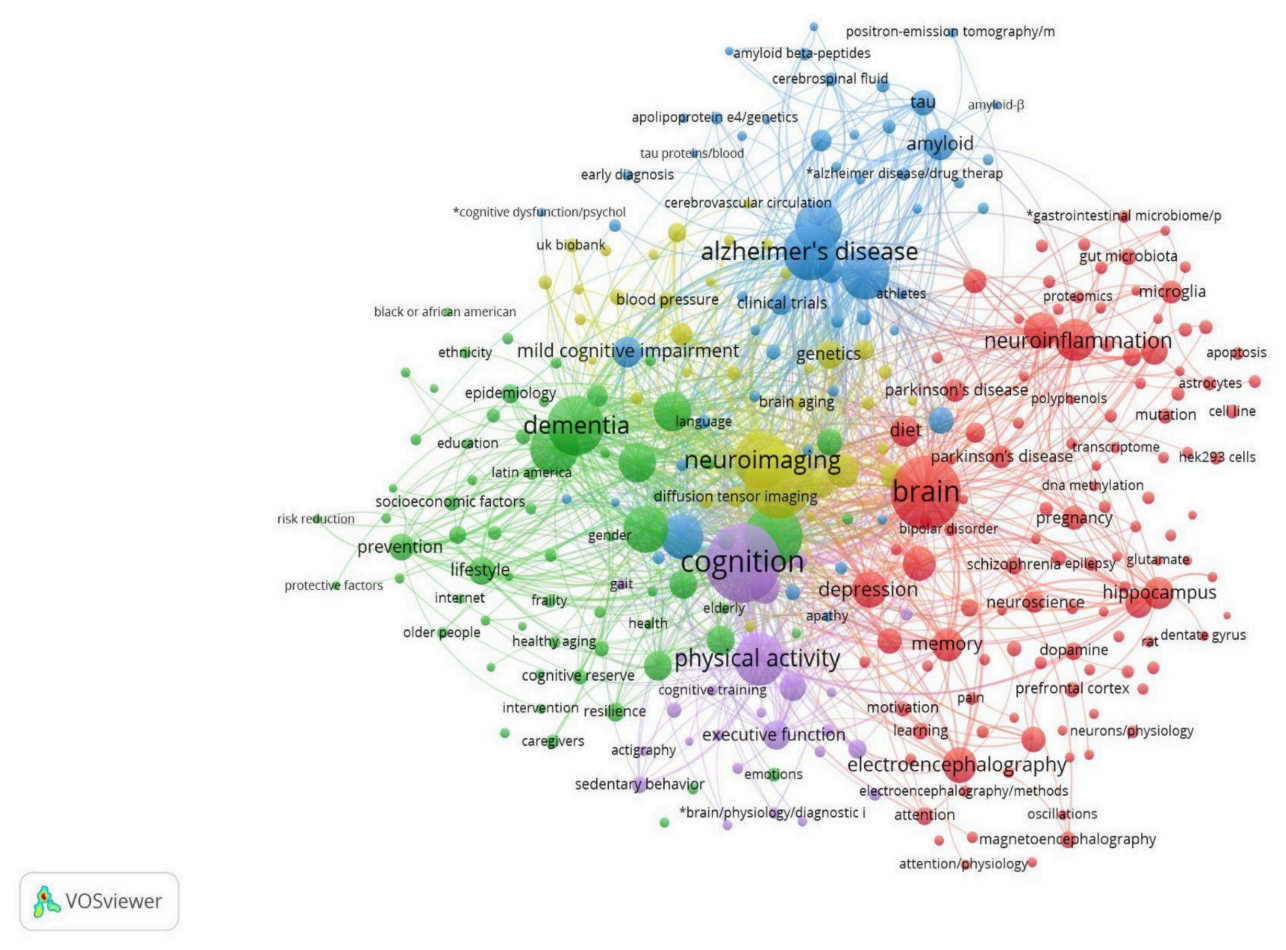
VOSviewer keyword co-occurrence network for the overall PubMed/MEDLINE corpus (1993–2026; *n* = 18,306). Node size represents keyword occurrence frequency, links represent co-occurrence relationships between keywords, and node colors indicate the five thematic clusters identified by VOSviewer. Keywords occurring at least 10 times were eligible for inclusion, and the 300 keywords with the highest total link strength were retained for network visualization and clustering.

A total of 56,210 keywords were identified across the 18,306 publications included in the analysis. Of these, 2138 met the minimum threshold of 10 occurrences. The 300 keywords with the highest total link strength were retained for network visualization and clustering. The analysis identified five thematic clusters representing the principal research domains within the analyzed network (Figure 2).

The five clusters differed in size and thematic focus (Table 1). The largest cluster (Cluster 1; *n* = 117) was centered on neurobiological and pathophysiological mechanisms, whereas Cluster 2 (*n* = 67) emphasized population brain health, cognition, lifestyle, and prevention. Cluster 3 (*n* = 54) was primarily associated with neurodegenerative diseases and biomarkers; Cluster 4 (*n* = 37) with neuroimaging, vascular health, and computational approaches; and Cluster 5 (*n* = 25) with neurophysiology, behavior, and cognitive interventions. Overall, the network therefore encompassed both biological and clinical domains and population-level, behavioral, and technological dimensions of brain health.

Across the complete network, cognition, brain, magnetic resonance imaging, aging, and dementia were among the most frequent and strongly connected keywords (Table 2). Cognition had the highest number of occurrences (*n* = 2136) and total link strength (7292). The term ‘brain health’ appeared in 779 publications, had a total link strength of 2375, and had an average publication year of 2023.03.

### 3.2. Temporal Overlay and Thematic Connections of the Term “Brain Health”

**Figure 3:**
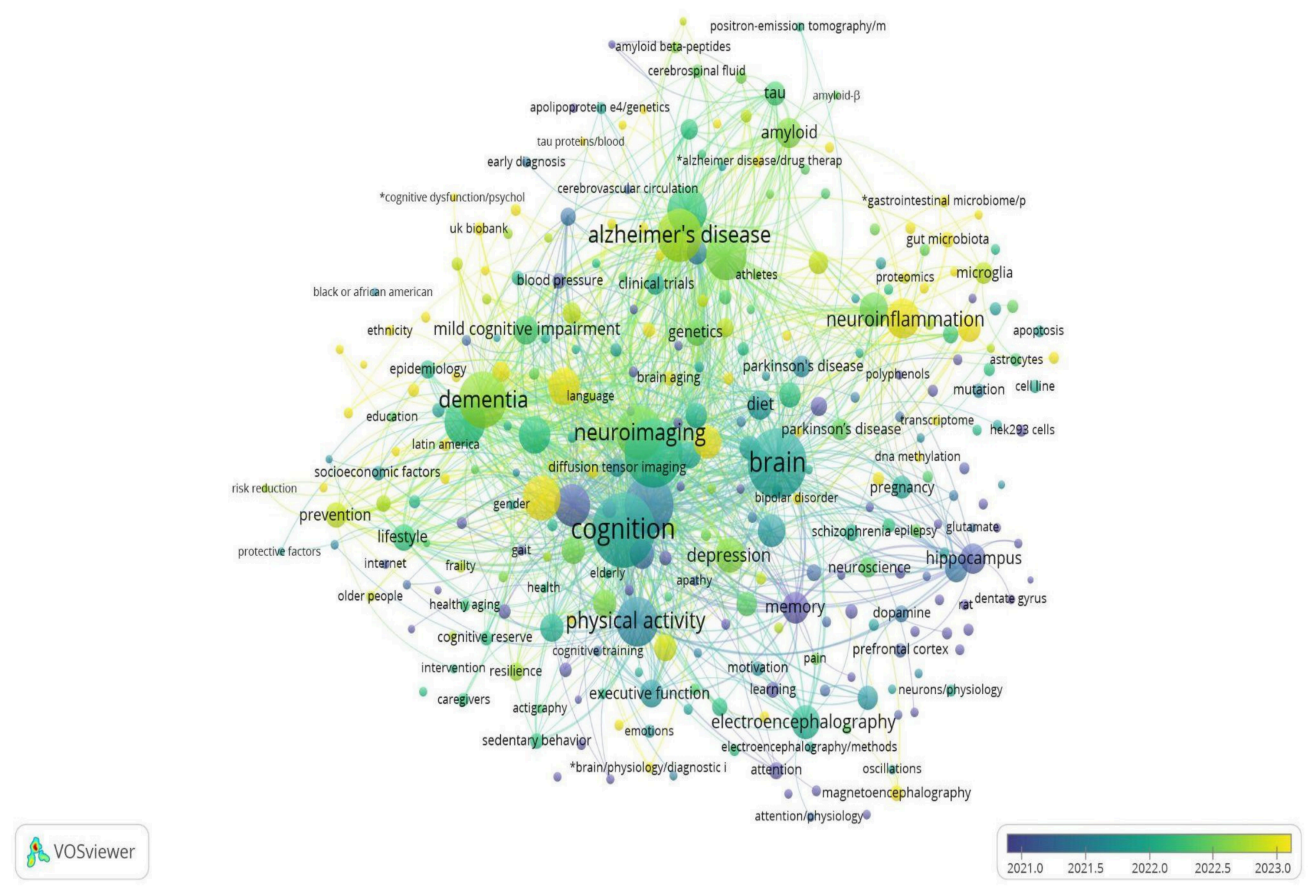
VOSviewer overlay visualization of the keyword co-occurrence network for the overall PubMed/MEDLINE corpus (1993–2026; *n* = 18,306). Node size represents keyword occurrence frequency, links represent co-occurrence relationships between keywords, and node color represents the average publication year associated with each keyword, with cooler colors indicating earlier and warmer colors indicating more recent average publication years within the displayed network. Keywords occurring at least 10 times were eligible for inclusion, and the 300 keywords with the highest total link strength were retained for visualization.

The overlay visualization of the co-occurrence map showed differences in the relative recency of keywords across the thematic structure, based on their average publication year (Figure 3).

The color scale represents the average publication year associated with each keyword, ranging approximately from 2021 (dark blue) to 2023 (bright yellow) within the displayed network; it does not represent the earliest and latest individual publications in the overall dataset. Keywords displayed in shades of yellow have relatively more recent average publication years and include terms related to neuroinflammation and the microbiome, such as neuroinflammation, gut microbiota, microglia, and proteomics.

These relatively recent areas include neuroinflammation, microbiome, artificial intelligence, and lifestyle interventions, indicating their prominence among topics with more recent average publication years in the analyzed corpus. In contrast, terms such as hippocampus, dopamine, and electroencephalography appear in cooler shades, indicating earlier average publication years within the displayed network. The term “brain health” itself had an average publication year of 2023.03, indicating a relatively recent temporal position within the analyzed keyword network.

**Figure 4:**
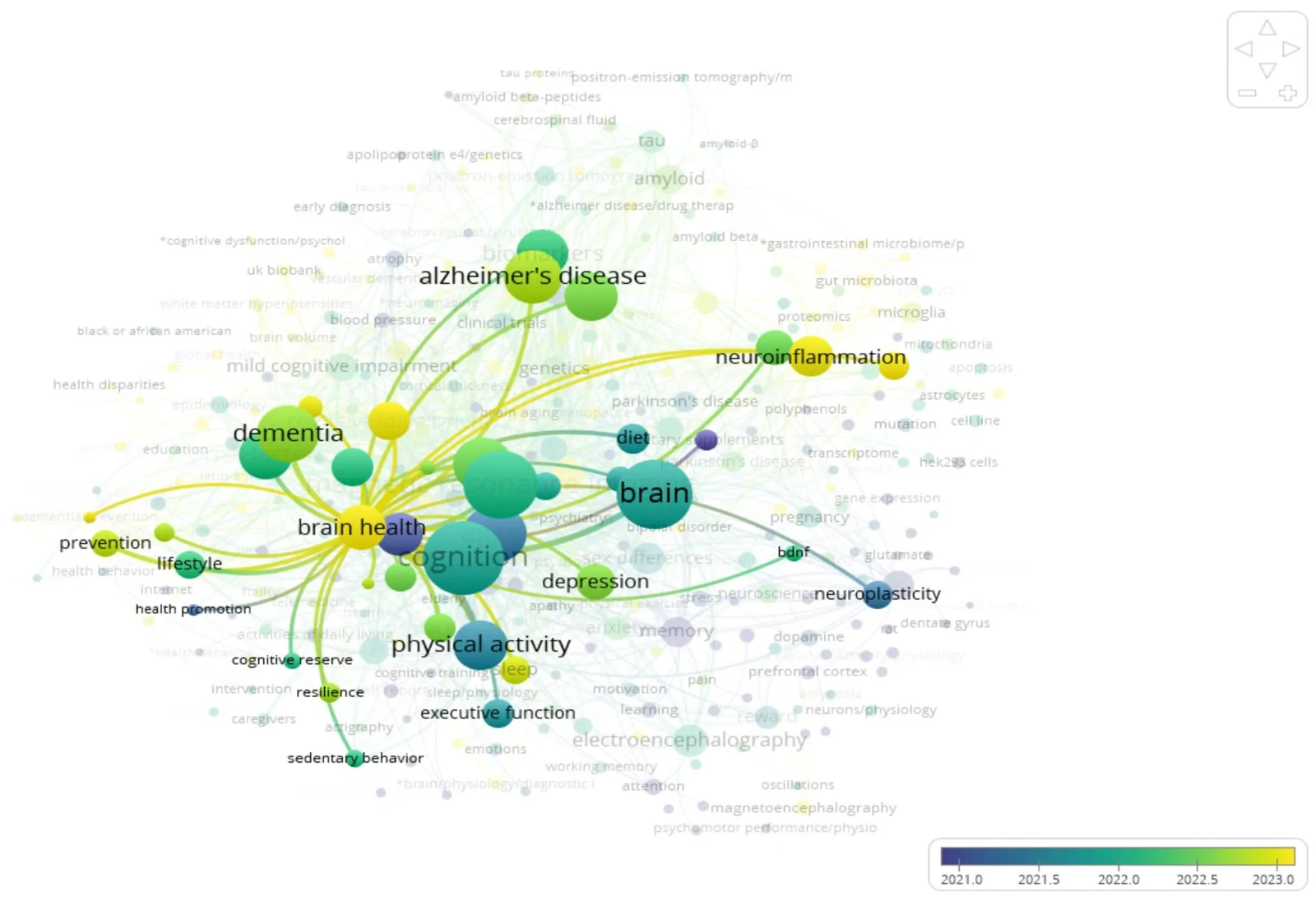
VOSviewer visualization of the direct keyword co-occurrence connections of the term “brain health” within the overall PubMed/MEDLINE corpus (1993–2026; *n* = 18,306). The visualization highlights the network relationships between “brain health” and directly connected terms across the analyzed keyword co-occurrence network.

Analysis of the direct connections of the term “brain health” (Figure 4) shows a rich network of interactions with concepts that cover both biological and social dimensions. The term “brain health” appears to function as a unifying interdisciplinary framework that connects neurobiology, public health, prevention, and lifestyle interventions.

Specifically, the term is strongly associated with cognition, brain, physical activity and dementia while also showing strong connections with lifestyle, prevention, depression and neuroplasticity. The presence of terms such as cognitive reserve, resilience and health promotion in the immediate network of "brain health" highlights the dual nature of the term: on the one hand as a biological state influenced by neurodegenerative processes and on the other hand as a dynamic concept linked to resilience and lifestyle interventions.

Overall, the temporal and structural characteristics of the network indicate that, within the analyzed PubMed/MEDLINE corpus, the term “brain health” is associated with keywords spanning molecular neurobiology, public health, and social medicine.

### 3.3. Targeted Analysis of Health-Economic Concepts

A targeted analysis of the complete dataset showed that health-economic concepts were present within the PubMed/MEDLINE corpus analyzed, although at relatively low frequencies. After consolidation of lexical and indexing variants, the most frequently identified concepts were cost–benefit analysis (*n* = 37), cost of illness (*n* = 28), cost-effectiveness (*n* = 24), and healthcare costs (*n* = 21), followed by economic evaluation and quality-adjusted life years (QALYs) (*n* = 15 each). Less frequently represented concepts included health expenditures (*n* = 9), health economics (*n* = 7), economic models (*n* = 6), economic burden (*n* = 4), and cost–utility analysis (*n* = 2), while return on investment was not identified. The complete results, including the lexical and indexing variants consolidated under each predefined concept, are reported in Supplementary Table S1. These findings indicate that economic-evaluation terminology was not absent from the underlying literature but remained relatively infrequent and did not achieve sufficient network prominence to appear among the 300 keywords with the highest total link strength retained for the primary co-occurrence visualization.

A supplementary VOSviewer co-occurrence analysis, using a minimum threshold of five occurrences and retaining up to 1000 keywords, confirmed that health-economic terms were represented within the broader keyword network but did not form a distinct thematic cluster. The occurrence counts reported in this supplementary VOSviewer network are not directly equivalent to the unique-publication counts reported in the targeted analysis. The targeted analysis counted unique publications containing each predefined health-economic concept after consolidation of lexical and indexing variants across the complete exported dataset, whereas the supplementary network reports VOSviewer occurrence values for standardized keyword terms after thesaurus and network processing. Accordingly, differences between these values, such as 37 unique publications versus 26 VOSviewer occurrences for cost–benefit analysis and 28 versus 19 for cost of illness, reflect differences in analytical procedure and unit of measurement rather than inconsistent counting. Cost–benefit analysis (occurrences = 26, TLS = 80) and cost of illness (occurrences = 19, TLS = 67), together with the related socioeconomic term caregiver burden (occurrences = 19, TLS = 77), were identified within Cluster 1. These terms were embedded within a broader clinical and patient-outcome-oriented cluster rather than forming a distinct health-economic grouping, demonstrating limited network prominence of economic-evaluation terminology within the analyzed PubMed/MEDLINE corpus.

### 3.4. Analysis of Recent Literature (2023–2026)

To characterize the contemporary thematic structure represented in the recent literature, a complementary descriptive bibliometric analysis was conducted on the independently retrieved recent-period corpus of 10,908 publications covering 2023 to the search date in 2026. This analysis was intended to characterize the thematic organization of the recent-period corpus on its own terms rather than to constitute a temporal subset analysis or a controlled longitudinal comparison with the overall corpus.

**Figure 5:**
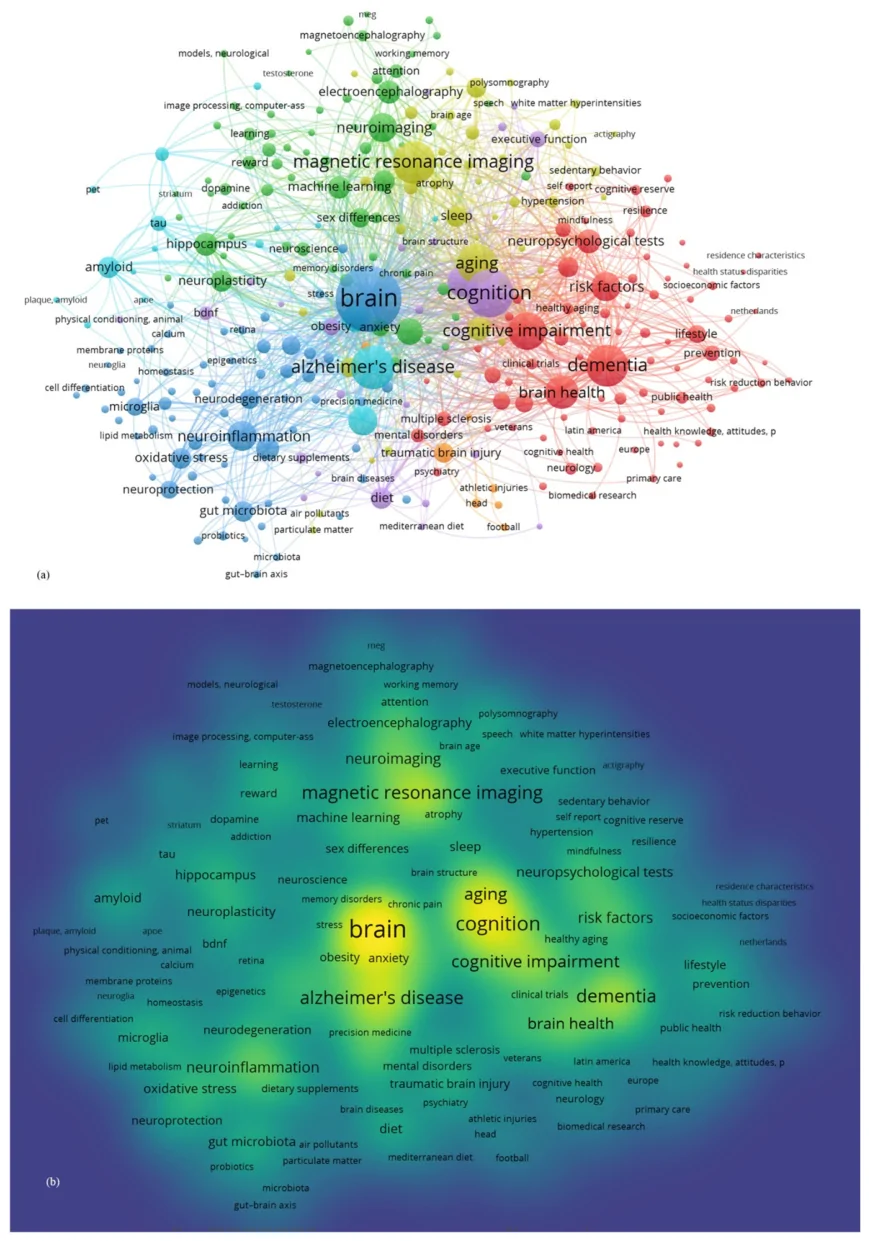
Bibliometric mapping of recent literature on brain health during the period 2023–2026. (**a**) Keyword co-occurrence network visualization, (**b**) density visualization of dominant research topics. In the density visualization, colors indicate keyword density, with yellow representing areas of higher keyword density and green-to-blue colors representing progressively lower density. Publications from 2026 represent a partial publication year because the recent-period search was conducted on 9 May 2026.

The same analytical parameters applied to the full dataset were retained for the recent-period analysis, including a minimum threshold of 10 occurrences and the selection of the 300 keywords with the highest total link strength (Figure 5).

The recent-period analysis identified seven thematic clusters, reflecting the thematic structure of this independently retrieved corpus (Table 3). The number and composition of these clusters are interpreted as descriptive characteristics of the recent-period network and are not used to infer temporal change relative to the overall corpus. Two domains emerged as distinct clusters in the recent network: neurodegenerative disease biomarkers and traumatic brain injury. Their separation from broader thematic groupings indicates that these topics were represented as distinct thematic domains within the recent-period network.

The recent-period analysis provided a complementary descriptive characterization of the thematic structure observed in the independently retrieved 2023–2026 corpus. It highlighted the prominence of blood-based biomarkers, neuroinflammation, precision medicine, early diagnosis, and other research directions within this recent-period network. Because this corpus was retrieved independently from the overall dataset and on a different search date, its network structure is interpreted descriptively and not as evidence of temporal change relative to the overall corpus. Because the search was completed before the end of 2026, publications from that year represent a partial publication year.

Within the independently retrieved recent-period corpus, the term “brain health” ranked 8th (occurrences = 600, TLS = 2176, avg. year = 2024.18). These values are reported as descriptive characteristics of the recent-period network and are not interpreted as evidence of temporal change relative to the independently retrieved overall corpus. At the same time, the term neuroinflammation (occurrences = 471, avg. year = 2024.45) was prominent in Cluster 3, alongside the terms gut microbiota, oxidative stress, and microglia, highlighting neuroinflammation as a prominent research area within the recent-period network.

## 4. Discussion

The bibliometric findings reveal a broad and differentiated thematic structure within the retrieved PubMed/MEDLINE brain health corpus, spanning molecular and neurobiological research, neurodegeneration and biomarkers, neuroimaging, lifestyle and prevention, and population-level perspectives. The following discussion interprets the principal patterns in relation to the broader literature while distinguishing these contextual interpretations from the findings generated directly by the bibliometric analysis. 

This study has several limitations. First, the analysis was restricted to PubMed/MEDLINE and may therefore underrepresent publications indexed primarily in economic, management, or multidisciplinary databases such as Scopus, Web of Science, and EconLit. This database restriction may partly contribute to the low representation and limited network prominence of health-economic concepts observed within the analyzed corpus. Second, bibliometric mapping depends on the terminology and keywords used in the original publications, which may result in inconsistent representation of emerging or insufficiently standardized concepts. In addition, restricting the network to the 300 keywords with the highest total link strength may have excluded less frequent but potentially relevant terms. Finally, the use of brain health as the sole search term provided a focused dataset but may not have captured studies using related terminology. In addition, the exact original record-level export corresponding to the independently retrieved recent-period corpus (*n* = 10,908) was not retained in a form permitting reliable reconstruction of its complete PMID list. Consequently, although the search strategy and retained VOSviewer analytical outputs are documented, exact record-level reproduction of the recent-period corpus is not possible. In addition, some VOSviewer normalization and clustering parameters, including any random seed, were not independently documented at the time of the original analysis and therefore cannot be reported with certainty. This limits the complete reproducibility of the network normalization and clustering procedure.

Accordingly, the observed low frequency and limited network prominence of health-economic terminology should be interpreted as characteristics of the corpus retrieved by the present search strategy and not as evidence of limited integration between brain health and health economics across the broader multidisciplinary literature. Future research should therefore extend the analysis across multiple databases and broader search strategies. The proposed brain health performance framework also requires prospective validation in national or regional healthcare settings. Further work should focus on developing standardized population-level brain health indicators and evaluating their usefulness for monitoring outcomes and informing resource allocation. Finally, the economic value of emerging blood-based biomarkers, including neurofilament proteins and GFAP, should be assessed through cost-effectiveness models that integrate diagnostic accuracy, clinical outcomes, and healthcare costs.

The principal bibliometric findings can be interpreted in relation to four broader themes discussed in the relevant literature. The first theme concerns the relatively recent prominence of neuroinflammation and the gut–brain axis. In the recent-period network, neuroinflammation (occurrences = 471, avg. year = 2024.45) and gut microbiota were represented among topics with relatively recent average publication years. In the broader literature, gut microbiota dysbiosis has been described as a potential mediator of neurodegenerative processes through the gut–brain axis, involving neuroinflammation, immunoregulation, pathological protein accumulation, and blood–brain barrier permeability; the relationship between gut microbiota and brain health has also been described as bidirectional and complex [26]. 

The second theme concerns the relatively recent representation of blood-based biomarkers. Terms such as neurofilament proteins (average publication year: 2025.10) and glial fibrillary acidic protein (GFAP; average publication year: 2025.04) showed particularly recent average publication years, highlighting less invasive biomarkers as a contemporary research focus in the analyzed corpus. Contextual evidence from the broader literature indicates that, while cerebrospinal fluid biomarkers remain among the best-studied fluid biomarkers for Alzheimer’s disease, recent research has also focused on less invasive blood-based biomarkers, including amyloid β42/40, phosphorylated tau, NFL, GFAP, and inflammatory markers [27]. A systematic review of 272 clinical trials in Alzheimer’s disease found that 44% used fluid biomarkers as endpoints, with blood and cerebrospinal fluid biomarkers used at approximately equal frequencies. The review also anticipated increasing use of blood biomarkers as the field moves toward primary prevention [28]. These findings are aligned with the revised criteria for the diagnosis and staging of Alzheimer’s disease, which incorporate blood biomarkers into the clinical–biological diagnostic and staging framework [29].

The third theme concerns the integration of machine learning in neuroimaging. The terms machine learning (avg. year = 2023.34) and UK Biobank (avg. year = 2024.12) showed relatively recent average publication years, indicating the contemporary representation of computational and large-scale data approaches within the analyzed corpus. As an example from the broader literature, the SPARE-Tau index was developed using machine-learning methods applied to flortaucipir PET data and was reported to detect pathology at early disease stages and predict progression compared with region-of-interest approaches [30]. At the same time, the research community recognizes the need for rigorous validation standards that ensure repeatability, interpretability, and computational efficiency of models before their clinical application [31]. Considered together with the broader literature, these bibliometric observations provide contextual support for exploring population-level brain health indicators as one component of the proposed conceptual framework.

The fourth theme concerns the limited prominence of health-economic concepts within the PubMed/MEDLINE brain health corpus analyzed in this study. The targeted analysis of the complete dataset showed that health-economic concepts were present, but at relatively low frequencies, with cost–benefit analysis, cost of illness, cost-effectiveness, and healthcare costs being the most frequently identified. Their absence from the primary 300-keyword co-occurrence network therefore reflects limited network prominence rather than absence from the underlying literature. This pattern becomes particularly relevant when considered alongside the more established use of economic evaluation and performance-monitoring approaches in specific neurological disorders. 

In the management of chronic neurological diseases, structured quality-monitoring approaches have already been implemented. For example, in Parkinson’s disease, a hub-and-spoke network in Lombardy demonstrated how structured organization using common diagnostic criteria and outcome measures can support patient management and facilitate research [32]. Similarly, in multiple sclerosis, a French framework comprising 48 quality-of-care indicators across seven domains—including MRI management and disease-modifying therapies—provides an example of systematic quality evaluation in chronic neurological disease [33]. In the economic domain, a multinational study of late-stage Parkinson’s disease reported three-month costs ranging from €12,156 in the United Kingdom to €25,649 in Sweden, with formal care, hospitalization, and informal care among the main contributors to costs [34]. This illustrates that documented costing and performance-monitoring approaches have been developed for specific neurological diseases and healthcare contexts, providing relevant contextual evidence for the conceptual framework proposed in this study. A related conceptual approach is the Brain Capital framework, which positions brain health and brain skills as productive assets that can be strengthened through investment and linked to broader economic and societal outcomes. Recent bibliometric evidence indicates that Brain Capital remains a young and relatively concentrated research field, with its neuroscience and brain-health foundations more developed than its economic, policy, and measurement dimensions. Importantly, the cited Brain Capital literature indicates that standardized and validated measurement frameworks remain at an early stage, particularly with respect to economic, policy, and measurement dimensions [35]. Taken together, the bibliometric findings indicate limited representation and network prominence of health-economic concepts within the PubMed/MEDLINE brain health corpus analyzed here. The literature discussed above additionally illustrates the relevance of performance measurement in specific neurological and health-system contexts; on this basis, performance management is incorporated as a conceptual component of the framework proposed in this study rather than as a separately quantified dimension of the targeted bibliometric analysis.

## 5. Proposed Conceptual Framework for the Economic and Performance Evaluation of Brain Health

A major finding of the bibliometric analysis was the limited representation and network prominence of health-economic concepts within the PubMed/MEDLINE brain health corpus analyzed in this study. The targeted analysis of the complete dataset identified several health-economic concepts, including cost–benefit analysis, cost of illness, cost-effectiveness, healthcare costs, economic evaluation, and quality-adjusted life years, although these occurred relatively infrequently and did not emerge among the 300 most strongly connected keywords in the primary co-occurrence network. Return on investment was not identified. Taken together, these findings indicate that health-economic concepts are present within the retrieved PubMed/MEDLINE corpus but show relatively low frequency and limited network prominence within the bibliometric structure analyzed in this study. For contextual comparison, previous bibliometric research in other medical fields has examined relationships between clinical research, healthcare costs, and management outcomes [20].

The limited prominence of health-economic concepts observed within the analyzed PubMed/MEDLINE corpus is particularly relevant given the substantial and increasing societal burden of neurological and neurodegenerative disorders. The global cost of dementia was estimated at US$1.3 trillion in 2019 and is projected to increase to approximately US$1.7 trillion by 2030, or almost US$2.8 trillion when corrected for expected increases in the costs of providing care, while the number of people living with dementia worldwide is projected to reach approximately 78 million by 2030 and 139 million by 2050 [36]. Within the European Union, the cost of neurodegenerative disease care has been projected to increase from EUR 267 billion in 2021 to EUR 528 billion by 2050, whereas effective lifestyle and risk-reduction interventions could potentially generate savings of up to EUR 558 billion [37]. These estimates demonstrate that brain health is not only a clinical and public health concern, but also a major economic and health-system priority.

Informed by the bibliometric findings and the broader literature discussed above, we therefore propose an interdisciplinary conceptual framework linking brain health with economic evaluation and health-system performance management. Performance management is incorporated in this framework as a conceptual and interpretive component informed by the broader literature; it was not assessed as a separately quantified dimension of the bibliometric analysis. The framework comprises three complementary axes: (1) measurement of the economic burden of neurodegenerative disease, (2) evaluation of the value and return on investment of preventive interventions, and (3) development of population-level brain health performance indicators. Together, these axes extend the concept of brain health from clinical description toward measurable health-system and societal outcomes. 

Axis 1: Economic Burden and Disease-Cost Assessment

The first axis focuses on the systematic measurement of the direct and indirect costs associated with neurodegenerative diseases. It builds primarily on the research domain represented by Alzheimer’s disease, mild cognitive impairment, amyloid, tau, biomarkers, and related diagnostic approaches. Although these areas were prominently represented in the analyzed PubMed/MEDLINE corpus, standardized costing and accounting terminology showed limited representation within the bibliometric evidence examined in this study.

Future research should develop cost-of-illness models stratified by disease stage, from mild cognitive impairment and early disease to moderate and advanced neurodegeneration. Such models should distinguish between direct medical costs, direct non-medical costs, informal caregiving, productivity losses, and wider societal consequences. They should also compare the costs of early diagnosis and monitoring with those associated with delayed detection, avoidable hospitalization, institutional care, and progressive loss of independence. This would allow the clinical value of biomarkers and diagnostic technologies to be considered alongside their economic and organizational consequences.

Axis 2: Economic Evaluation and Return on Investment of Prevention

The second axis concerns the economic evaluation of preventive and risk-reduction strategies. It draws on the cluster linking brain health with physical activity, lifestyle, diet, prevention, healthy aging, and other modifiable determinants. These findings indicate that prevention is a prominent component of the analyzed PubMed/MEDLINE corpus, whereas health-economic concepts related to the value of preventive strategies showed limited representation within the bibliometric evidence examined in this study. 

Evidence suggests that lifestyle-based interventions may reduce the risk or delay the onset of neurodegenerative disease. For example, adherence to the Mediterranean diet has been associated with a lower risk of early Parkinson’s disease [38], while non-pharmacological interventions across the European Union may produce substantial long-term savings [37]. Building on this evidence, future studies should evaluate the cost-effectiveness, cost–benefit profile, and return on investment of prevention programs implemented in communities, workplaces, and healthcare systems.

Relevant outcomes could include healthcare costs avoided, cases of cognitive impairment delayed or prevented, quality-adjusted life-years gained, preservation of functional independence, reduced caregiver burden, and improvements in productivity. In workplace settings, the evaluation could additionally incorporate absenteeism, presenteeism, disability, and early retirement. This would enable preventive brain health interventions to be assessed according to their broader organizational and societal value.

Axis 3: Population-Level Brain Health Performance Indicators

The third axis focuses on the development of standardized indicators for monitoring brain health at the population and health-system levels. It builds on research involving neuroimaging, machine learning, genetics, large-scale datasets, and resources such as the UK Biobank. The use of computational methods and population databases creates opportunities to integrate biological, cognitive, behavioral, and social information into multidimensional measures of brain health. 

Possible applications include the development of a brain health index and predictive models linking brain health indicators with future healthcare utilization and costs. Such indicators could combine cognitive performance, mental health, physical activity, vascular risk, functional independence, social participation, digital measures, blood-based biomarkers, and selected neuroimaging parameters. 

At the health-system level, brain health key performance indicators could assess access to preventive services, timeliness of diagnosis, functional outcomes, caregiver support, and disparities across socioeconomic or geographical groups. However, these indicators would require prospective validation, standardized definitions, and careful assessment of feasibility, equity, privacy, and cost before implementation.

**Figure 6:**
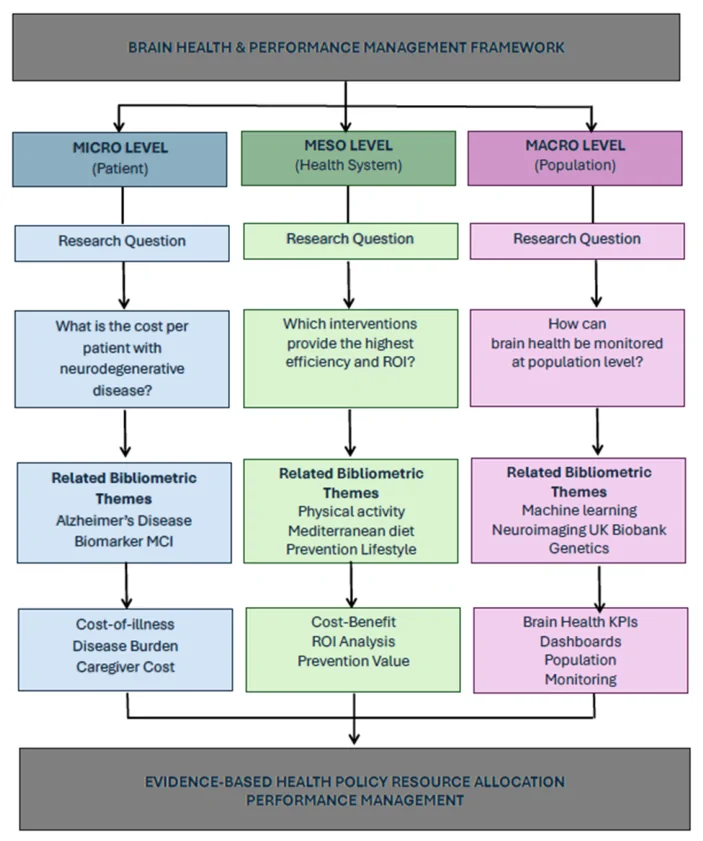
Proposed conceptual Brain Health and Performance Management Framework informed by the bibliometric findings and the broader literature discussed in the manuscript. Blue boxes represent the micro (patient) level, green boxes the meso (health-system) level, and purple boxes the macro (population) level; gray boxes denote the overarching framework and its evidence-based policy and resource-allocation integration.

Overall, the proposed framework connects three levels of analysis: the individual level, through disease burden and patient-related costs; the intervention level, through economic evaluation and return on investment; and the population level, through standardized performance indicators and monitoring systems. Its structure and proposed measures are summarized in Figure 6.

## 6. Conclusions

The present bibliometric analysis identified a broad and differentiated thematic structure in the overall 1993–2026 network. The independently retrieved recent-period 2023–2026 corpus was analyzed separately and showed a seven-cluster thematic structure in which topics such as neuroinflammation, blood-based biomarkers, and machine-learning applications were prominently represented. Because the two corpora were retrieved independently on different search dates, the recent-period network is interpreted descriptively rather than as evidence of temporal change relative to the overall network. Average publication year is likewise interpreted only as an indicator of the relative recency of individual topics.

Within the analyzed PubMed/MEDLINE corpus, health-economic concepts occurred at relatively low frequencies and showed limited prominence in the principal keyword co-occurrence network. The targeted analysis nevertheless identified several health-economic concepts in the complete dataset, while the supplementary co-occurrence analysis showed that these terms were embedded within broader thematic structures rather than forming a distinct health-economic cluster.

These findings should be interpreted within the scope of the adopted search strategy. The analysis was restricted to PubMed/MEDLINE and used the exact search expression “brain health”; therefore, the findings characterize the retrieved corpus and should not be generalized to the broader multidisciplinary brain-health literature.

Informed by these bibliometric findings and the broader literature discussed in the manuscript, the proposed conceptual three-axis framework integrates disease-cost assessment, economic evaluation and return-on-investment assessment of preventive interventions, and population-level brain health performance indicators. The framework should be regarded as a conceptual proposal requiring prospective validation; in particular, its performance-management component was not assessed as a separately quantified dimension of the bibliometric analysis.

## Figures and Tables

**Table 1:** Thematic clusters identified in the keyword co-occurrence analysis of the overall PubMed/MEDLINE corpus (1993–2026; *n* = 18,306).

Cluster	Items, n	Proposed Thematic Label	Representative Keywords	Interpretive Focus
1.	117.	Neurobiological mechanisms and pathophysiology	brain; neuroinflammation; hippocampus; memory; depression; dopamine; neurodegeneration; neuroplasticity; oxidative stress	Molecular, cellular, and functional mechanisms underlying brain function, neurological disorders, and neuropsychiatric symptoms.
2.	67.	Population brain health, cognition, and lifestyle	cognition; dementia; physical activity; aging; prevention; lifestyle; brain health; healthy aging; risk factors; longitudinal studies	Population-level determinants of brain health, with emphasis on aging, cognitive outcomes, modifiable risk factors, and prevention.
3.	54.	Neurodegenerative diseases and biomarkers	Alzheimer’s disease; amyloid; tau; biomarkers; mild cognitive impairment; neuropsychological tests; cerebrospinal fluid; positron emission tomography; plasma biomarkers; frontotemporal dementia	Diagnosis and characterization of neurodegenerative diseases using cognitive assessments, fluid biomarkers, and molecular imaging.
4.	37.	Neuroimaging, vascular health, and computational analysis	magnetic resonance imaging; neuroimaging; diffusion tensor imaging; brain aging; genetics; white matter; stroke; machine learning; UK Biobank; cerebrovascular disease	Structural and vascular determinants of brain health investigated through neuroimaging, population datasets, genetics, and computational methods.
5.	25.	Neurophysiology, behavior, and cognitive interventions	executive function; electroencephalography; sleep; sedentary behavior; cognitive training; working memory; functional connectivity; gait; actigraphy	Neurophysiological and behavioral assessment of cognition, sleep, mobility, and the effects of lifestyle or cognitive interventions.

Note: Cluster labels were assigned by the authors on the basis of the most frequent and strongly connected keywords within each cluster. The listed terms are representative rather than exhaustive. Publications from 2026 represent a partial publication year because the overall search was conducted on 8 April 2026.

**Table 2:** Most frequent and strongly connected keywords in the overall PubMed/MEDLINE corpus (1993–2026; *n* = 18,306).

Rank	Keyword	Occurrences (n)	Total Link Strength	Average Publication Year
1.	Cognition	2., 136	7., 292	2021. 87
2.	Brain	1., 863	5., 086	2021. 85
3.	Magnetic resonance imaging	1., 838	5., 789	2022. 10
4.	Aging	1., 309	4., 413	2021. 40
5.	Dementia	1., 251	4., 258	2022. 62
6.	Neuroimaging	1., 130	3., 850	2022. 48
7.	Alzheimer’s disease	1., 104	3., 737	2022. 70
8.	Physical activity	931.	3., 204	2021. 54
9.	Risk factors	889.	2., 892	2022. 18
10.	Biomarkers	840.	2., 904	2022. 21
11.	Brain health	779.	2., 375	2023. 03

Note: Occurrences indicate the number of publications in which each keyword appeared. Total link strength represents the cumulative strength of a keyword’s co-occurrence. Average publication year indicates the mean publication year of the publications associated with each keyword. Publications from 2026 represent a partial publication year because the overall search was conducted on 8 April 2026.

**Table 3:** Thematic clusters identified in the keyword co-occurrence analysis of the recent-period PubMed/MEDLINE corpus (2023–2026; *n* = 10,908), based on the 300 keywords with the highest total link strength retained for network visualization and clustering.

Cluster	Items (n)	Thematic Interpretation	Representative Central Terms
1.	81.	Public Health, Dementia & Interventions	dementia; cognitive impairment; brain health; risk factors; neuropsychological tests; longitudinal studies; mental health; lifestyle
2.	64.	Neuroimaging & Neurophysiology	neuroimaging; depression; hippocampus; electroencephalography; machine learning; neuroplasticity; memory; sex differences
3.	64.	Neurobiological Mechanisms & Neuroinflammation	brain; neuroinflammation; neurodegenerative diseases; gut microbiota; oxidative stress; neurodegeneration; Parkinson’s disease; microglia
4.	42.	Cerebral Vascular Health & MRI	magnetic resonance imaging; aging; stroke; sleep; genetics; white matter; gray matter; cerebrovascular circulation
5.	25.	Cognitive Function & Lifestyle	cognition; physical activity; diet; obesity; executive function; BDNF; dietary supplements; cardiorespiratory fitness
6.	17.	Biomarkers of Neurodegeneration	Alzheimer’s disease; biomarkers; amyloid; tau; positron-emission tomography; cerebrospinal fluid
7.	7.	Traumatic Brain Injury	traumatic brain injury; brain injuries; athletic injuries; football; athletes

Note: Publications from 2026 represent a partial publication year because the recent-period search was conducted on 9 May 2026.

## Data Availability

The bibliographic data analyzed in this study were retrieved from the publicly accessible PubMed/MEDLINE database. The retained materials supporting reproducibility of the analyses, including the original PubMed bibliographic export batches from the overall search, the complete documented search strategy, the customized VOSviewer thesaurus, retained VOSviewer analytical output files, documented analytical parameters, and source data for the study flow diagram, are provided with the article as Supplementary Source Files. The exact original record-level export corresponding to the recent-period analytical corpus (*n* = 10,908) was not retained in a form that permits reliable reconstruction of its complete PMID list; therefore, no retrospective PubMed retrieval has been substituted for the original dataset.

## Supplementary Materials

The following supporting information can be downloaded at: https://www.mdpi.com/article/10.3390/brainsci16090967/s1, Table S1: Occurrence of predefined health-economic concepts in the complete brain health dataset (1993–2026); Table S2: Annual publication counts in the overall analytical PubMed/MEDLINE corpus (1993–2026); Supplementary Source Files S1–S5: Original PubMed bibliographic export batches retained from the overall search conducted on 8 April 2026; Supplementary Source File S6: Customized VOSviewer thesaurus file; Supplementary Source File S7: Retained VOSviewer analytical output for the overall bibliometric network; Supplementary Source File S8: Retained VOSviewer analytical output for the recent-period bibliometric network; Supplementary Source File S9: Retained VOSviewer analytical output for the supplementary health-economic network analysis; Supplementary Source File S10: Documented VOSviewer analytical parameters; Supplementary Source File S11: Documented PubMed/MEDLINE search strategy for the overall and recent-period searches; Supplementary Source File S12: Source data for the study flow diagram (Figure 1).

## References

[1] Harerimana B., Forchuk C., Walsh J., Fogarty J., Borrie M. (2021). Brain Health: A Concept Analysis. Issues Ment. Health Nurs..

[2] Chen Y., Demnitz N., Yamamoto S., Yaffe K., Lawlor B., Leroi I. (2022). Defining brain health: A concept analysis. Int. J. Geriatr. Psychiatry.

[3] Pilatti L.D.S., Borges C.R., Apóstolos-Pereira S.L., Blood M.R.Y., Castilhos R.M., Batista B.L.L., de Moraes F.M., Lange M.C. (2025). Brain health: A new concept to the neurologist—An initiative of the Lifestyle Medicine Commission of the Brazilian Academy of Neurology. Arq. Neuropsiquiatr..

[4] Nicotra A., Maestri G., Salvadori E., Pantoni L. (2024). Brain health assessment. An exploratory review of tools related to its cognitive dimension. Cereb. Circ. Cogn. Behav..

[5] Cavaco M.A., Jang S.R., Olsen C., Bodnar C., Ferko N. (2025). Global Societal Burden of Alzheimer’s Disease by Severity: A Targeted Literature Review. Neurol. Ther..

[6] Chaudhuri K.R., Azulay J.-P., Odin P., Lindvall S., Domingos J., Alobaidi A., Kandukuri P.L., Chaudhari V.S., Parra J.C., Yamazaki T. (2024). Economic Burden of Parkinson’s Disease: A Multinational, Real-World, Cost-of-Illness Study. Drugs Real World Outcomes.

[7] Mudrazija S., Aranda M.P., Gaskin D.J., Monroe S., Richard P. (2025). Economic Burden of Alzheimer Disease and Related Dementias by Race and Ethnicity, 2020 to 2060. JAMA Netw. Open.

[8] Huang Y., Li X., Liu Z., Huo J., Guo J., Chen Y., Chen Y., Chen R. (2022). Projections of the economic burden of care for individuals with dementia in mainland China from 2010 to 2050. PLoS ONE.

[9] Yılmaz S., Boz C., Özsarı S.H., Yılmaz F., Türkön B.F., Mete A.H. (2025). Effects of Neurological Disorders on Health Expenditure and Economic Output: Dynamic Panel Analysis for OECD Countries. Systems.

[10] Li W., Kim K.-W.R., Zhang D., Liu B., Dengler-Crish C.M., Wen M., Shi L., Pan X., Gu Y., Li Y. (2023). Cost-effectiveness of physical activity interventions for prevention and management of cognitive decline and dementia-a systematic review. Alzheimers Res. Ther..

[11] Eaglestone G., Gkaintatzi E., Jiang H., Stoner C., Pacella R., McCrone P. (2023). Cost-Effectiveness of Non-pharmacological Interventions for Mild Cognitive Impairment and Dementia: A Systematic Review of Economic Evaluations and a Review of Reviews. PharmacoEconomics Open.

[12] Braun A., Höfler M., Auer S. (2024). Cost-Effectiveness of Prevention for People at Risk for Dementia: A Scoping Review and Qualitative Synthesis. J. Prev. Alzheimers Dis..

[13] Walsh S., Brain J., Mukadam N., Anderson R., Greene L., Govia I., Kuhn I., Anstey K.J., Knapp M., Stephan B.C. (2022). A systematic review of the cost-effectiveness of community and population interventions to reduce the modifiable risk factors for dementia. Maturitas.

[14] Ritchie C.W., Waymont J., Pennington C., Draper K., Borthwick A., Fullerton N., Chantler M., Porteous M., Danso S., Green A. (2022). The Scottish Brain Health Service Model: Rationale and Scientific Basis for a National Care Pathway of Brain Health Services in Scotland. J. Prev. Alzheimers Dis..

[15] García-García I., Donica O., Cohen A.A., Nusslé S.G., Heini A., Nusslé S., Pichard C., Rietschel E., Tanackovic G., Folli S. (2023). Maintaining brain health across the lifespan. Neurosci. Biobehav. Rev..

[16] Yang Y., Lv C., Li H., Chen K., Li X., Chen Y., Zhang J., Wei D., Lu P., Wang J. (2021). Community-based Model for Dementia Risk Screening: The Beijing Aging Brain Rejuvenation Initiative (BABRI) Brain Health System. J. Am. Med. Dir. Assoc..

[17] Zhu Z., Wang H., Zhang L., Zeng L., Gan L., Jia B. (2025). Advances in flexible sensor monitoring technology for neurodegenerative diseases in the elderly. Nanotechnology.

[18] Cruz M.V., Jamal S., Sethuraman S.C. (2025). A comprehensive survey of brain–computer interface technology in health care: Research perspectives. J. Med. Signals Sens..

[19] Galitskaya V., Polydoros G., Antoniou A.-S., Pergantis P., Drigas A. (2026). Brain–Computer Interfaces in Learning Disorders and Mathematical Learning: A Scoping Review with Structured Narrative Synthesis. Appl. Sci..

[20] Lampropoulos I.C., Malli F., Aggelopoulos E., Tsameti A., Rouka E., Daniil Z., Gourgoulianis K.I. (2025). Mapping the Scientific Landscape Between Respiratory Conditions and Costs: A Bibliometric Analysis. Healthcare.

[21] Van Eck N.J., Waltman L. (2010). Software survey: VOSviewer, a computer program for bibliometric mapping. Scientometrics.

[22] Lampropoulos I.C., Kalogera M. (2026). A Bibliometric Analysis of Performance Measurement and Management in Child and Adolescent Healthcare Services. Healthcare.

[23] Smyrnova-Trybulska E., Morze N., Kuzminska O., Kommers P. (2018). Mapping and visualization: Selected examples of international research networks. J. Inf. Commun. Ethics Soc..

[24] Bukar U.A., Sayeed S., Razak S.F.A., Yogarayan S., Amodu O.A., Mahmood R.A.R. (2023). A method for analyzing text using VOSviewer. MethodsX.

[25] van Eck N.J., Waltman L., Ding Y., Rousseau R., Wolfram D. (2014). Visualizing Bibliometric Networks. Measuring Scholarly Impact.

[26] Tarawneh R., Penhos E. (2022). The gut microbiome and Alzheimer’s disease: Complex and bidirectional interactions. Neurosci. Biobehav. Rev..

[27] Varesi A., Carrara A., Pires V.G., Floris V., Pierella E., Savioli G., Prasad S., Esposito C., Ricevuti G., Chirumbolo S. (2022). Blood-Based Biomarkers for Alzheimer’s Disease Diagnosis and Progression: An Overview. Cells.

[28] Oosthoek M., Vermunt L., de Wilde A., Bongers B., Antwi-Berko D., Scheltens P., van Bokhoven P., Vijverberg E.G.B., Teunissen C.E. (2024). Utilization of fluid-based biomarkers as endpoints in disease-modifying clinical trials for Alzheimer’s disease: A systematic review. Alzheimers Res. Ther..

[29] Jack C.R., Andrews J.S., Beach T.G., Buracchio T., Dunn B., Graf A., Hansson O., Ho C., Jagust W., McDade E. (2024). Revised criteria for diagnosis and staging of Alzheimer’s disease: Alzheimer’s Association Workgroup. Alzheimers Dement..

[30] Toledo J.B., Rashid T., Liu H., Launer L., Shaw L.M., Heckbert S.R., Weiner M., Seshadri S., Habes M., for the Alzheimer’s Disease Neuroimaging Initiative (2022). SPARE-Tau: A flortaucipir machine-learning derived early predictor of cognitive decline. PLoS ONE.

[31] Leenings R., Winter N.R., Dannlowski U., Hahn T. (2022). Recommendations for machine learning benchmarks in neuroimaging. NeuroImage.

[32] Albanese A., Di Fonzo A., Fetoni V., Franzini A., Gennuso M., Molini G., Pacchetti C., Priori A., Riboldazzi G., Volonté M.A. (2020). Design and Operation of the Lombardy Parkinson’s Disease Network. Front. Neurol..

[33] Veillard D., Deburghgraeve V., Le Page E., Debouverie M., Wiertlewski S., Gallien P., Edan G. (2022). Developing tools to evaluate quality of care management for patients living with multiple sclerosis: An original French initiative. Rev. Neurol..

[34] Kruse C., Lipinski A., Verheyen M., Balzer-Geldsetzer M., Wittenberg M., Lorenzl S., Richinger C., Schmotz C., Tönges L., Woitalla D. (2024). Care of Late-Stage Parkinsonism: Resource Utilization of the Disease in Five European Countries. Mov. Disord..

[35] Iddouch K. (2026). Brain Capital at the Frontier of Knowledge A Systematic Literature Review and Bibliometric Analysis of an Emerging Transdisciplinary Fields. https://papers.ssrn.com/sol3/papers.cfm?abstract_id=6685158.

[36] World Health Organization (2021). Global Status Report on the Public Health Response to Dementia.

[37] Maresova P., Rezny L., Bauer P., Valko M., Kuca K. (2024). Nonpharmacological intervention therapies for dementia: Potential break-even intervention price and savings for selected risk factors in the European healthcare system. BMC Public Health.

[38] Maraki M.I., Yannakoulia M., Xiromerisiou G., Stefanis L., Charisis S., Giagkou N., Kosmidis M.H., Dardiotis E., Hadjigeorgiou G.M., Sakka P. (2023). Mediterranean diet is associated with a lower probability of prodromal Parkinson’s disease and risk for Parkinson’s disease/dementia with Lewy bodies: A longitudinal study. Eur. J. Neurol..

