# Supplementary material for: Brain Health Research in PubMed/MEDLINE: A Bibliometric Analysis of Thematic Structure and Health-Economic Terminology with a Conceptual Performance-Management Framework
Source: Brain Sci. 2026 Sep 13;16(9):967. doi: 10.3390/brainsci16090967 (PMC13604335; doi:10.3390/brainsci16090967)
Supplement: Supplementary file 1 [file brainsci-16-00967-s001.zip › S1.pdf]

Table S1. Occurrence of predefined health-economic concepts in the complete brain health dataset (1993–2026).

**Predefined health-economic concept**	**Representative lexical/indexing variants consolidated**	**Publications, n**
Cost–benefit analysis	cost-benefit analysis; cost benefit analysis	**37**
Cost of illness	cost of illness; cost-of-illness	**28**
Cost-effectiveness	cost-effectiveness; cost effectiveness; cost-effectiveness analysis; cost effectiveness analysis	**24**
Healthcare costs	health care costs; healthcare costs; healthcare cost; health-care costs	**21**
Economic evaluation	economic evaluation	**15**
Quality-adjusted life years (QALYs)	quality-adjusted life years; QALYs	**15**
Health expenditures	health expenditure; health expenditures	**9**
Health economics	health economics; brain health economics; economics, medical	**7**
Economic models	economic models; models, economic; economic modeling	**6**
Economic burden	economic burden	**4**
Cost–utility analysis	cost-utility analysis; cost utility analysis; cost-utility	**2**
Return on investment	return on investment; ROI	**0**

Note: Counts represent unique publications containing each predefined concept after consolidation of lexical and indexing variants. Variants differing only in capitalization, hyphenation, singular/plural form, or PubMed indexing format were grouped under the corresponding concept. A publication containing multiple variants of the same concept was counted once for that concept. Socioeconomic descriptors were not classified as health-economic evaluation concepts.

Table S2. Annual publication counts in the overall analytical PubMed/MEDLINE corpus (1993–2026; n = 18,306).

Year	Publications, n
1993	1
1994	0
1995	0
1996	0
1997	0
1998	0
1999	1
2000	1
2001	8
2002	9
2003	4
2004	2
2005	12
2006	11
2007	21
2008	19
2009	38
2010	37
2011	51
2012	70
2013	111
2014	167
2015	274
2016	399
2017	521
2018	687
2019	894
2020	1,222
2021	1,607
2022	1,902
2023	2,106
2024	2,713
2025	3,714
2026	1,704
**Total**	**18,306**

Note: Annual counts were derived from the final bibliographic dataset used for the overall VOSviewer analysis. Publications from 2026 represent a partial publication year because the overall search was conducted on 8 April 2026.
